# Age-Related Changes in the Global DNA Methylation Profile of Leukocytes Are Linked to Nutrition but Are Not Associated with the MTHFR C677T Genotype or to Functional Capacities

**DOI:** 10.1371/journal.pone.0052570

**Published:** 2012-12-20

**Authors:** Marcus V. M. Gomes, Leandro V. Toffoli, Douglas W. Arruda, Larissa M. Soldera, Gislaine G. Pelosi, Rejane D. Neves-Souza, Eliane R. Freitas, Denilson T. Castro, Audrey S. Marquez

**Affiliations:** 1 Research Centre on Health Sciences, University of Northern Parana (UNOPAR), Londrina, Paraná, Brazil; 2 Centre of Biological Sciences, Department of Physiological Sciences, State University of Londrina (UEL), Londrina, Parana, Brazil; Sapporo Medical University, Japan

## Abstract

Global DNA methylation of peripheral blood leukocytes has been recently proposed as a potential biomarker for disease risk. However, the amplitude of the changes in DNA methylation associated with normal aging and the impacts of environmental changes on this variation are still unclear. In this context, we evaluated the association of global DNA methylation with nutritional habits, tobacco smoking, body mass index (BMI), clinical laboratory parameters, polymorphism C677T MTHFR, functional cognition and the daily practice of physical activity in a cancer-free older population. Leukocyte global DNA methylation from 126 older individuals was quantified using a high-throughput ELISA-based method. Global DNA hypomethylation was observed in older individuals when compared to a younger population (p = 0.0469), confirming changes in DNA methylation in the aging process. Furthermore, the methylation profile of elders was correlated with the daily ingestion of carbohydrates (p = 0.0494), lipids (p = 0.0494), vitamin B6 (p = 0.0421), magnesium (p = 0.0302), and also to the serum levels of total protein (p = 0.0004), alpha 2 globulin (p = 0.0013) and albumin (p = 0.0015). No statistically significant difference was observed when global DNA methylation were stratified according to C677T MTHFR genotypes (p = 0.7200), BMI (p = 0.1170), smoking habit (p = 0.4382), physical activity in daily life (p = 0.8492), scored cognitive function (p = 0.7229) or depression state (p = 0.8301). Our data indicate that age-related variations in the global DNA methylation profile of leukocytes might be modulated by the daily intake of carbohydrates, lipids, vitamin B6, and magnesium and be associated with serum protein levels, however it is independent of C677T MTHFR genotype and not correlated with BMI, smoking habit, cognitive function or the routine physical activities.

## Introduction

DNA methylation of cytosine generally followed by guanine (CpG nucleotides) is the most widely studied epigenetic marker, with comprehensive implications in the control of embryo development, cell differentiation, X chromosome inactivation in females, genomic imprinting, suppression of endogenous retroviruses and chromosomal stability [1]–[6].

Studies from the last decades have strongly associated abnormalities in DNA methylation patterns with the etiology of various diseases, including cancer, cardiovascular diseases, pediatric syndromes, autoimmune diseases and genetic disorders [7]. Moreover, recent data have pointed to the potential for using global DNA methylation profiles of peripheral blood leukocytes as a suitable biomarker for diseases risk, especially for cancer [8]–[11].

In parallel, a growing number of reports have pointed to a direct implication of changes in DNA methylation profiles in the aging process. Global DNA hypomethylation [12] and *loci* specific hypermethylation of the promoter regions of various genes, including *LOX*, *p16INK4a*, *RUNX3*, *TIG1*, *RASSF1A*, *GSTP1*, *ESR1* and others were previously associated with normal aging [13]–[16].

Intriguingly, the two best-known epigenetic alterations “global DNA hypomethylation” and “loci-specific hypermethylation” are frequently described in cancer, indicating a possible molecular connection between the age-related accumulation of epigenetic alterations and malignant transformation [16]–[18]. However, whether age-associated changes in DNA methylation profiles are consequent to the aging process or to exogenous environmental exposures is still unclear.

Hence, we aimed to evaluate in the present study the association of age-related changes in the global DNA methylation profile of leukocytes to environmental factors in a cancer free older population. Furthermore, we explored whether the range of variation of global DNA methylation profiles is associated with the polymorphism C677T of the methylenetetrahydrofolate reductase (MTHFR), BMI, clinical laboratory values, functional cognition and the capacity for physical activity.

## Materials and Methods

### Ethics Statement

As approved by the Research Ethics Committee of the University of Northern Parana (UNOPAR), a comprehensive questionnaire was administered and written informed consent obtained from all the participants.

### Study Participants

The studied population comprised 126 physically independent men and women, aged 60–88 years at baseline, participating in the thematic project “Study of Aging and Longevity (EELO)”. The EELO project consists of an ongoing population study, performed in Londrina city, Parana State - Brazil, focused on the determination of epidemiologic profiles, social-demographic parameters and regional health related indicators for the older population.

Physical independency status of older individuals was considered the major inclusion criterion and it was obtained according to the classification proposed by the Functional Status Spirduso (levels 3 and 4). This means that an older individual is able to perform basic activities of daily life and also the instrumental activities of daily life. The individuals from level 3 have low exercise capacity and are sedentary, and individuals from level 4 have above average exercise capacity and are considered as physically active [19].

A group consisting of 33 healthy young volunteers (18 men and 15 women) aged 18–35 was considered as the control population for molecular analysis. Patients with a history of cancer or familiar metabolic disturbance were excluded from both control and patient groups.

### DNA Sampling, Global DNA Methylation Profile and MTHFR Genotyping

Venous blood samples were collected at baseline and leukocyte DNA was obtained by the salting out method [20].

Global DNA methylation was determined by dosage of methyl (CH_3_) using the Imprint Methylated DNA Quantification Kit – IMDQ1 (Sigma-Aldrich) as previously described [21]. The MDQ1 is a high-throughput, molecular biology kit, which uses a 96-well plate format to provide accurate differential global DNA methylation. Methylation status of each sample was calculated by the amount of methylated cytosines in the sample (5 mC) relative to global cytidine (5 mC+dC) in a positive control (100% methylated) that had been previously methylated and a no template control sample (0% methylated), using absorbance readings at 450 nm and following the formula: (A450sample-A450 NTC)/(A450met - A450 NTC)×100. All samples were analyzed in triplicate.

One of the most extensively studied genetic polymorphisms that can modify the availability of methyl groups in folate mediated one-carbon metabolism and affects the DNA methylation process is the C677T polymorphism of the gene that codes for the enzyme methylenetetrahydrofolate reductase (MTHFR). In order to test the association of the MTHFR-C677T polymorphism and the global methylation profile all the elders were genotyped by the Restriction Fragment Length Polymorphism (RFLP) approach using the Hinf I enzyme for fragmentation of PCR amplicons.

### Food Intake and Tobacco Smoking

We analyzed the total energy intake and the amount and proportions of macronutrients (proteins, carbohydrates, lipids) and micronutrients (B6 and B12 vitamins, folic acid, magnesium and zinc) of elders by the dietary 24-hour recall method [22]. The interviews were conducted on three different days, taking one day on the weekend and two mid-week. With the aid of a photo album with pictures of portion sizes and food, the interviews took place with notation of the food consumed in the order of daily meals. The types of food, the quantities consumed and how they were prepared were registered. The quantities of these foods were reported in household measures and converted into grams or milliliters. Dietary data were processed and analyzed with the nutritional evaluation software Avanutri online [23].

The prevalence of present and past tobacco smoking was evaluated according to the Heatherton classification for smoking habits [24].

### Blood Tests

Clinical laboratory values were determined by standard biochemical automatic or semi-automatic methods. Serum total cholesterol, LDL, HDL and triglycerides were quantified by Automatic Enzymatic Colorimetry (AU400 Beckman Coulter, Brea, CA). Glucose, urea, creatinine, and uric acid were quantified using Semi-Automatic Enzymatic Colorimetric Equipment (Bioplus®Bio2000). Alanine transaminase (ALT), aspartate transaminase (AST), and alkaline phosphatase were quantified with the AU400 Automated Kinetic Analyzer (Beckman Coulter, USA). Levels of thyroxine (T4), Triiodothyronine (T3) and thyroid-stimulating hormone (TSH) were measured by Chemiluminescence (Abbott Architects, USA). Total protein, alpha-1-globulin, beta-1-globulin, beta-2-globulin and gamma-globulin were quantified by capillary electrophoresis (Minicap Sebia, France).

### Physical Activity in Daily Life

The level of PADL (physical activity in daily life) was objectively measured by analysis of the average of one week of steps/day using a pedometer (Yamax SW-200 Digiwalker®, Japan). The elderly were instructed to attach the pedometer to the right side of the waist as soon as waking up, to reset the device display and to wear it for at least 12 hours/day for one week. They were also strictly instructed to maintain their usual routine and to not wear the device only for sleeping and taking baths. In addition, each day the subjects had to fill out a diary recording the time of starting and finishing the PADL measurement as well as registering the number of steps counted at the end of the day.

### Mini Mental State Examination

For this study we used the Mini Mental State Examination Questionnaire (MMSE) [25], adapted for the Brazilian population [26]. The MMSE is an important tool in screening for cognitive impairment, which consists of questions related to temporal orientation, spatial immediate memory, calculation, recall, naming and repetition of words, command of an action, reading, preparation of a sentence and copying a drawing. The MMSE score may range from a minimum of 0 points, which indicates the highest degree of cognitive impairment of individuals to a total maximum of 30 points, which, in turn, corresponds to better cognitive ability. The cutoff points used were those suggested by Murden et al. [27], 24 for people with education above 9 years and 17 for those with less education.

### State of Depression

Depression levels were assessed by the Beck Depression Inventory (BDI) [28], adapted to Portuguese. The BDI consists of a validated questionnaire with 21 questions that measure the degree of depression in elderly subjects. A score of 0–12 points indicates a person without clinical depression; from 13 to 20 indicates mild depressive symptoms; 21 to 30 indicates moderate depression and 31 or more severe depression.

### Statistical Analysis

Age associated changes in DNA methylation were assessed by comparison of the mean percentage of global DNA methylation of older individuals and a young healthy population by the Mann Whitney test. The Kruskal-Wallis Test was used for the stratified analysis of DNA methylation according to gender in both older and young groups.

Correlations between percentage of global DNA methylation of leukocytes and food intake, BMI, tobacco smoking status, clinical laboratory values, PADL, and scored cognition and depression state were assessed by the non-parametric Spearman coefficient test. Stratification of global DNA methylation according to the MTHFR C677T polymorphism was performed by One-way ANOVA and Tukey’s Multiple Comparison Test. There was no missing data for any of our outcome variables. p-values less than 0.05 were considered statistically significant. All analyses were computed using Prism GraphPad 5.0 software.

## Results

**Figure 1:**
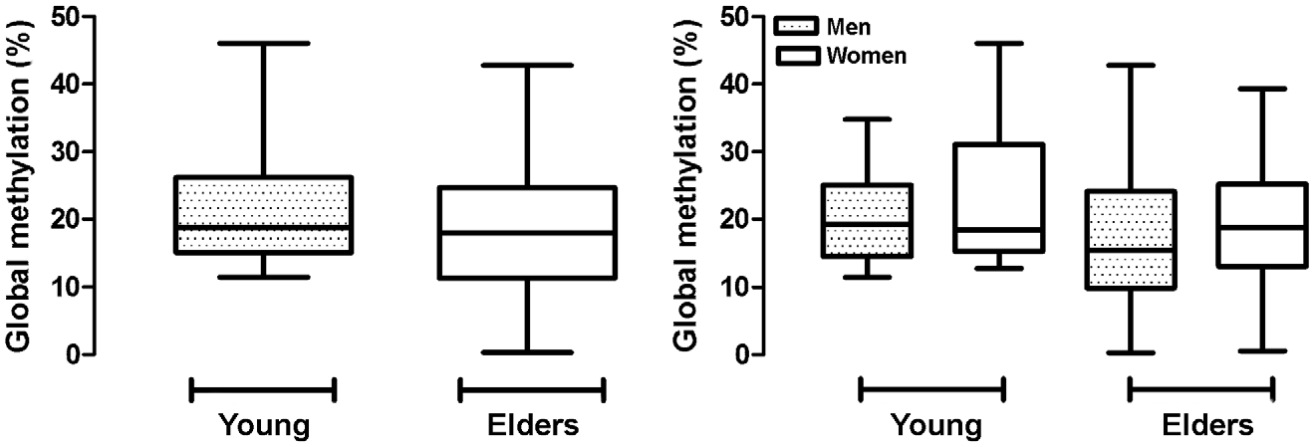
Graphic representation of the age-related global DNA hypomethylation. A) Percentage of Global DNA Methylation of leukocytes from young and elder individuals. B) Stratification of global DNA methylation according to gender in both young and older groups.

Characteristics of the studied population are depicted in Table 1. The comparative analyses of the mean percentage of global DNA methylation of leukocytes from elders (mean = 18.17; 95%CI, 16.57, 19.77) and young healthy individuals (mean = 21.88; 95%CI, 18.88, 24.88) revealed a statistically significant difference (p = 0.0469), confirming previous evidence of the involvement of a decrease in the global DNA methylation profile in aging. No statistical association was observed when data were stratified according to gender in both older and younger groups (p = 0.0915) (Figure 1).

**Table 1:** Characteristics of the studied population.

Characteristics	Total sample(126)	95% CI
Age (years)	70.8	69.66–71.89
Female sex (%)	52.4	**–**
BMI (kg/m^2^)	27.8	27.03–28.58
Current smoker (%)	9.5	**–**
MTHFR C677T genotype (%) (CC/CT/TT)	57/31/12	**–**
Global DNA methylation (%)	18.17	16.57–19.77

**Figure 2:**
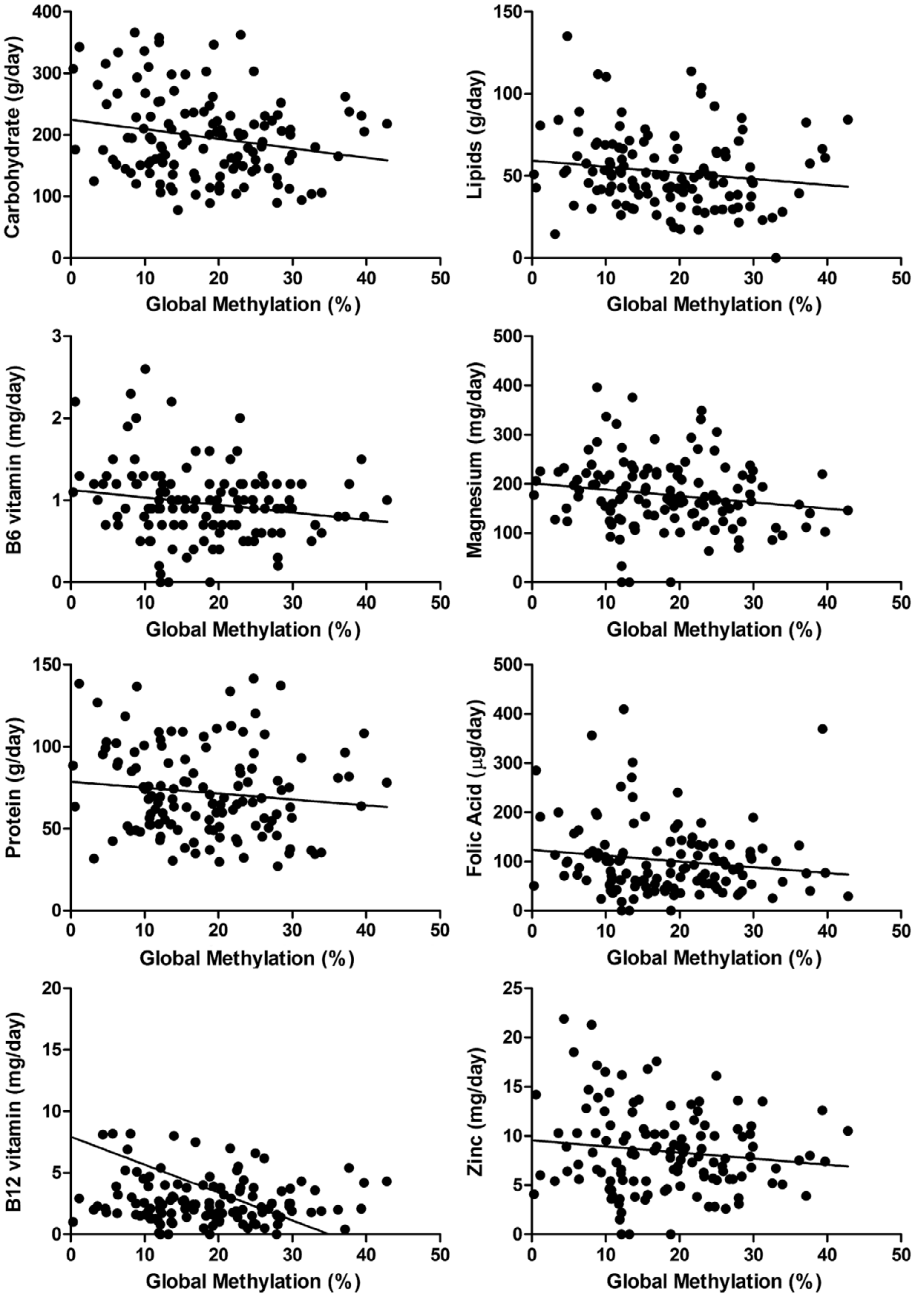
Correlations between global DNA methylation and nutritional profile. Daily intake of A) carbohydrate, B) Lipids, C) Vitamin B6, D) Magnesium, E) Protein, F) Folic Acid), G) Vitamin B12, H) Zinc.

No statistically significant difference was observed when global DNA methylation was stratified according to C677T MTHFR genotypes (p = 0.7200).The mean global DNA methylation of the elderly population showed a statistical association with daily ingestion of carbohydrates (p = 0.0494), lipids (p = 0.0494), vitamin B6 (p = 0.0421), and magnesium (p = 0.0302) (Figure 2). No statistically significant difference was observed when mean DNA methylation was correlated to daily ingestion of protein (p = 0.1359), folic acid (p = 0.0926), vitamin B12 (p = 0.2690) or zinc (p = 0.2918) (Table 2) (Figure 2). Furthermore, there was no statistically significant correlation between global DNA methylation of leukocytes and body mass index (p = 0.1170) or tobacco smoking habits (p = 0.4382) (Table 2).

**Table 2:** Correlations between global DNA methylation, Age, BMI, Smoking and Nutrition.

		Correlation with Global DNA Methylation		
Characteristic	Mean (SD)	95% CI	*p* value	*Spearman* coefficient (r)
Age	70.77 (+/−0.5629)	−0.2427 to 0.1158	0.4659	−0.06554
BMI	27.8 (+/−0.3900)	−0.04068 to 0.3125	0.1170	0.1404
Smoking	–	−0.2485 to 0.1126	0.4382	−0.07025
Protein (g/day)	72.13 (+/−2.390)	−0.3062 to 0.04758	0.1359	−0.1336
Carbohydrate (g/day)	196.51 (+/−6.068)	−0.3446 to 0.004728	0.0494	−0.1754
Lipid (g/day)	52.56 (+/−2.009)	−0.3550 to −0.007120	0.0361	−0.1869
Folic Acid (µg/day)	102.5 (+/−6.539)	−0.3218 to 0.03036	0.0926	−0.1505
B6 Vitamin (mg/day)	0.9603 (+/−0.04043)	−0.3500 to −0.001422	0.0421	−0.1814
**B12Vitamin (µg/day)**	**3.820 (+/−0.8854)**	−**0.2743 to 0.08226**	**0.2690**	−**0.09922**
**Magnesium (mg/day)**	**177.9 (+/−6.304)**	−**0.3607 to** −**0.01366**	**0.0302**	−**0.1932**
**Zinc (mg/day)**	**8.435 (0.3804)**	−**0.2701 to 0.08684**	**0.2918**	−**0.09465**

**Figure 3:**
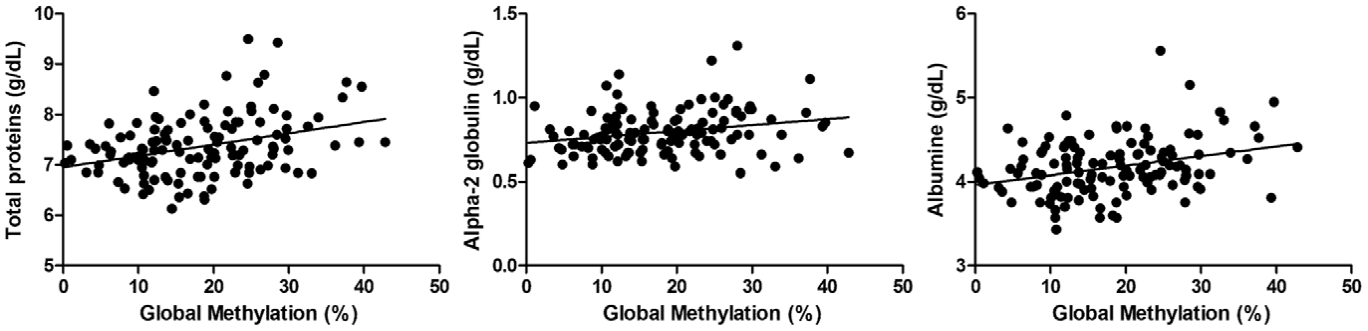
Correlations between global DNA methylation and serum dosages of proteins, albumin and alpha2 globulin.

When compared to clinical laboratory values, the DNA methylation profile of leukocytes showed a statistical correlation to the mean level of total protein (p = 0.0004*), alpha 2 globulin* (p = 0.0013) and albumin (p = 0.0015) (Table 3) (Figure 3). However, DNA methylation was not significantly correlated with other clinical laboratory parameters as demonstrated in Table 3.

**Table 3:** Correlation between global DNA methylation and clinical laboratorial values.

		Correlation with Global DNA Methylation		
Clinical laboratorial parameter	Mean (SD)	95% CI	*p* value	*Spearman* coefficient (r)
Total Cholesterol (mg/dL)	199.2 (+/−3.266)	−0.08220 a 0.2744	0.2687	0.09928
HDL Cholesterol (mg/dL)	56.17 (+/−1.029)	−0.1898 a 0.1702	0.9103	−0.01014
LDL (mg/dL)	113.4 (+/−2.502)	−0.08182 a 0.2747	0.2669	0.09966
Triglycerides (mg/dL)	147.8 (+/−8.326)	−0.1266 a 0.2324	0.5430	0.05469
Urea (mg/dL)	36.83 (+/−1.095)	−0.2251 a 0.1342	0.6016	−0.04696
Creatinine (mg/dL)	0.899 (+/−0.02481)	−0.3387 a 0.01140	0.0586	−0.1690
Uric Acid (mg/dL)	4.766 (+/−0.1236)	−0.3226 a 0.02938	0.0905	−0.1514
Glucose (mg/dL)	106.2 (+/−0.1236)	−0.07293 a 0.2830	0.2265	0.1085
ALT (mg/dL)	19.17 (+/−0.8257)	−0.2327 a 0.1263	0.5408	−0.05499
AST (mg/dL)	16.53 (+/−0.8942)	−0.2274 a 0.1318	0.5827	−0.04941
Alkaline Phosphatase (mg/dL)	60.76 (+/−1.522)	−0.1648 a 0.1952	0.8614	0.01571
Total Proteins (g/dL)	7.359 (+/−0.05351)	0.1360 a 0.4628	0.0004	0.3084
Albumin (g/dL)	4.169 (+/−0.02966)	0.1047 a 0.4374	0.0015	0.2794
Alpha1 globulin (g/dL)	0.3084 (+/−0.0041)	−0.1376 a 0.2217	0.6289	0.04346
Alpha 2 globulin (g/dL)	0.7950 (+/−0.0115)	0.1081 a 0.4403	0.0013	0.2826
Beta 1 globulin (g/dL)	0.4618 (+/−0.0078)	−0.01191 a 0.3382	0.0593	0.1685
Beta 2 globulin (g/dL)	0.394 (+/−0.0087)	−0.04534 a 0.3083	0.1295	0.1358
Gamma glob (g/dL)	1.226 (+/−0.0291)	−0.06028 a 0.2946	0.1770	0.1210
Hb A1C (%)	6.279 (+/−0.1005)	−0.1548 a 0.2050	0.7727	0.02598
Total T3 (ηg/mL)	1.308 (+/−0.0278)	−0.09439 a 0.2630	0.3321	0.08710
Free T4 (ηg/mL)	1.203 (+/−0.0199)	−0.1294 a 0.2297	0.5642	0.05185
TSH (µIU/mL)	2.750 (+/−0.2500)	−0.1387 a 0.2207	0.6374	0.04240

**Figure 4:**
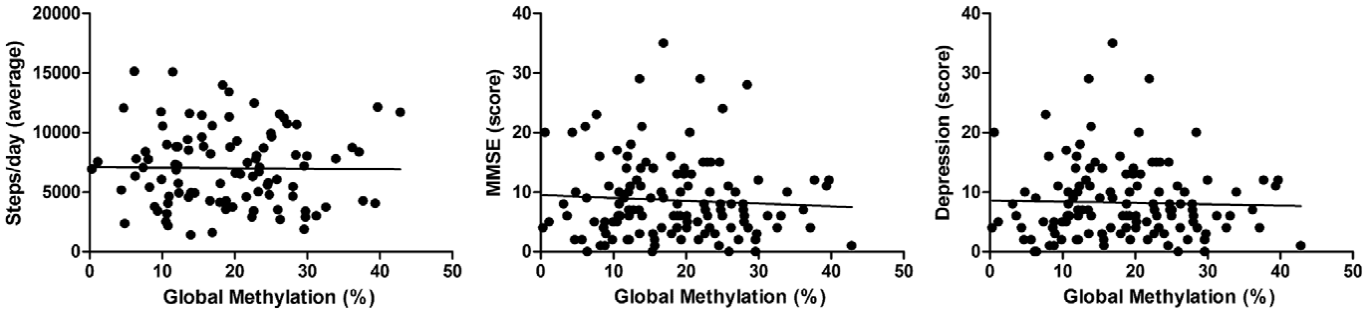
Representation of correlations between global DNA methylation, mean steps/day and scored functional cognition.

No statistically significant difference was observed between the DNA methylation profile of elders and the PADL (p = 0.8492), the media of scored cognition state (p = 0.7229) or depression state (p = 0.8301) (Table 4) (Figure 4).

**Table 4:** Correlation of global DNA methylation with scored cognition and physical activity.

		Correlation with Global DNA Methylation		
Characteristic	Mean (SD)	95% CI	*p* value	*Spearman* coefficient (r)
Media steps/day	7.016 (+/−325.6)	−0.2227 a 0.1855	0.8492	−0.01946
Scored Depression State (BDI)	8.216 (+/−0.5570)	−0.1619 a 0.1994	0.8301	0.01939
Scored Cognition State (MMSE)	8.643 (+/−0.5947)	−0.2107 a 0.1490	0.7229	−0.03190

## Discussion

Rapidly expanding knowledge related to life-long cellular-environmental interactions has been observed during the last decades. However, the molecular mechanisms by which the environment sensitizes cells and its effects on human health and aging are not completely understood.

Recent studies on the vulnerability of epigenetic mechanisms to environmental changes have provided substantial information in this regard. Increasing data have revealed the potential of some environmental factors, such as chemical pollutants, dietary components and other exogenous factors, to modulate the establishment and maintenance of epigenetic modifications, thereby leading to long-lasting effects [29].

In the present study, we showed that age-related changes in the global DNA methylation profile of leukocytes are vulnerable to nutritional factors, in particular the daily intake of carbohydrates, lipids, vitamin B6, and magnesium. Furthermore, our data showed an association between the DNA methylation profile of leukocytes and the serum levels of total proteins, alpha-2-globulin and albumin, and also demonstrated that the age-related changes in DNA methylation are independent of the polymorphism C677T of the MTHFR gene.

First evidences in animal models suggested that dietary intake of micronutrients involved in the one-carbon metabolism, namely folate, vitamin B12, vitamin B6, methionine, choline and betaine might be associated to the availability of the methyl donor S-adenosylmethionine (SAM) and consequently change the DNA methylation patterns [30], however the precise mechanisms by which nutrition affects global DNA methylation are not completely understood and increasing data in humans have refuted the previous simplistic hypothesis [31], [32].

Additionally, recent studies involving caloric restriction (CR) models have provided interesting data about the molecular mechanisms through which nutrition might affects epigenetic mechanisms and aging [33], [34]. Notable, CR has been shown to potentially affect the activity of DNA methyltransferases [35], [36] thereby modulating the DNA methylation mechanisms. However, future studies on this field are needed to further clarify the reasons why nutrition induces global DNA methylation changes in older individuals.

The functional relationship between epigenetic modifications and aging is still unknown, although the relationship between specific epigenotypes and disease phenotypes has been thoroughly studied [7], [37].

The association between decreasing global DNA methylation and aging has been reported in corroborating studies involving both human and animals models [12], [38]–[40]. Fundamental evidences of the involvement of epigenetics in aging were earlier provided by Fraga *et al.* in a study involving different-aged monozygotic (MZ) twins. By demonstrating that elderly MZ twin pairs present more epigenetic differences than young phenotypically similar MZ twin pairs, this study contributed to the notion that differential environmental exposures impact epigenetic inter-individualities and might implicate phenotypic divergence over time, often observed for example with respect to the differential susceptibility of MZ twins to common diseases [41].

A more recent age-related study in twins combined the concept of epigenetic vulnerability at locus specific regions to environmental changes and the aging process. By analyzing the DNA methylation of the promoter regions of the dopamine receptor 4 gene (*DRD4*), the serotonin transporter gene (*SLC6A4/SERT*) and the X-linked monoamine oxidase A gene (*MAOA*) in DNA samples at both ages 5 and 10 years in 46 MZ twin-pairs and 45 DZ twin-pairs, Wong *et al* showed that locus specific methylation differences are apparent already in early childhood, even between genetically identical individuals, and that the individual differences in methylation are not stable over time [42].

Global DNA hypomethylation in blood cells has been recently considered as an important biomarker for cancer risk, with reported implications for colorectal adenoma [43]–[44], head and neck squamous cell carcinoma [45], bladder cancer [46], [47], [10], breast cancer [9], gastric cancer [48]–[49] and renal cancer [8]. Furthermore, the lower levels of global DNA methylation observed later in life in adults with cancer might also be present early in life in children with a family history of cancer [50].

Several population studies have shown an association between the C677T polymorphism of the methylenetetrahydrofolate reductase gene (MTHFR), that codes for an enzyme required for folate metabolism and the generation of methyl groups, with global changes in DNA methylation [51], [52]. In the present study the C677T MTHFR polymorphism was not associated to global DNA methylation changes in older individuals. Moreover, the possibility of associations with other genetic variants cannot be excluded.

In a recent study Bell *et al* [53] provided indications using genome-wide approaches that in a small set of genes DNA methylation may be a candidate mechanism of mediating not only environmental, but also genetic effects on age-related phenotypes. However, additional studies are needed to further clarify the vulnerability of loci specific methylation to the nutritional profile and whether or not site specific methylation of the age-related genes shows similar relationship to global DNA methylation as well.

There is accumulating evidence suggesting that physical activity may affect epigenetic mechanisms and exert a protective effect against disease. By analyzing the global DNA methylation profile of peripheral blood from a cancer-free population aged 45–75 years Zhang et al [54] demonstrated that individuals with physical activity of 26–30 min/day had a significantly higher level of global genomic DNA methylation compared to those with physical activity ≤10 min/day, although the association became statistically insignificant after adjusting for age, gender, and other lifestyle factors.

In the present study we addressed whether global DNA methylation in elders is associated with the routine practice of moderate physical activity by measuring the average of one week of steps/day using a pedometer. No evidence of a statistically significant association was observed in our study.

The implication of abnormal methylation patterns and the etiology of aging-related diseases have also been proposed during the last decades [55]–[56]. Alzheimer’s and Type-2 diabetes (TD2) are two examples of non-oncogenic diseases strongly associated with aging that have been found to be associated with specific epigenetic alterations. Amyloid-b protein deposition in the aged brain of individuals with Alzheimer disease and abnormal expression of the *COX7A1* gene, which is involved in glucose metabolism, were previously associated with DNA demethylation [57] and increased age –associated methylation [58], respectively.

Furthermore, epigenetic changes have been recently proposed to be associated with the decline of cognitive function that normally occurs during aging [59].

A lack of association between the global DNA methylation profile of leukocytes and cognitive abilities of older individuals was found in the present study. Corroborating data was recently demonstrated by Schiepers et al [60]. In a study composed of a population of 215 men and women, aged 50–70, the global DNA methylation profile of leukocytes was not associated with cognitive function in the domains of memory, sensorimotor speed, complex speed, information speed and word fluency. Taking together, these recent reports do not support the association of changes in global DNA methylation with age-related cognitive dysfunction as previously hypothesized [59]. However, brain specific or genomic loci specific changes in methylation cannot be excluded in both studies. Future data are needed to further clarify the involvement of epigenetics in age related cognitive dysfunction.

In summary, our data indicate that the decreased global DNA methylation profile of leukocytes of older individuals might be associated with the daily intake of carbohydrates, lipids, vitamin B6, and magnesium, and also with serum protein levels, leading us to speculate that the nutritional profile might exert an important role in the aging process.

Furthermore, we showed that age-related changes in DNA methylation are independent of the MTHFR C677T polymorphism, body mass index or smoking habit and are not correlated with cognitive functions or the routine practicing of moderate physical activity.
